# Lymphocyte homing and recirculation with tumor tertiary lymphoid structure formation: predictions for successful cancer immunotherapy

**DOI:** 10.3389/fimmu.2024.1403578

**Published:** 2024-07-15

**Authors:** Weihong Tian, Wangzhi Wei, Gaofeng Qin, Xuanwen Bao, Xuecheng Tong, Min Zhou, Yuan Xue, Yu Zhang, Qixiang Shao

**Affiliations:** ^1^ Department of Immunology, School of Medicine, Jiangsu University, Zhenjiang, Jiangsu, China; ^2^ Life Science Institute, Jinzhou Medical University, Jinzhou, Liaoning, China; ^3^ Department of Medical Oncology, The First Affiliated Hospital, School of Medicine, Zhejiang University & Key Laboratory of Cancer Prevention and Intervention, Ministry of Education, Hangzhou, Zhejiang, China; ^4^ Changzhou Third People’s Hospital, Changzhou Medical Center, Nanjing Medical University, Changzhou, Jiangsu, China; ^5^ Institute of Medical Genetics and Reproductive Immunity, School of Medical Science and Laboratory Medicine, Jiangsu College of Nursing, Huai’an, Jiangsu, China

**Keywords:** lymphocyte homing, adhesion, C-X-C chemokines, dendritic cells, tertiary lymphoid structures, immunotherapy

## Abstract

The capacity of lymphocytes continuously home to lymphoid structures is remarkable for cancer immunosurveillance and immunotherapy. Lymphocyte homing and recirculation within the tumor microenvironment (TME) are now understood to be adaptive processes that are regulated by specialized cytokines and adhesion molecule signaling cascades. Restricted lymphocyte infiltration and recirculation have emerged as key mechanisms contributing to poor responses in cancer immunotherapies like chimeric antigen receptor (CAR)-T cell therapy and immune checkpoint blockades (ICBs). Uncovering the kinetics of lymphocytes in tumor infiltration and circulation is crucial for improving immunotherapies. In this review, we discuss the current insights into the adhesive and migrative molecules involved in lymphocyte homing and transmigration. The potential mechanisms within the TME that restrain lymphocyte infiltration are also summarized. Advanced on these, we outline the determinates for tertiary lymphoid structures (TLSs) formation within tumors, placing high expectations on the prognostic values of TLSs as therapeutic targets in malignancies.

## 1 Introduction

The effectiveness of cancer immunotherapy significantly relies on the infiltration of functional tumor-associated lymphocytes (1, 2). Systemic lymphocyte circulation involves a cyclical process in which lymphocytes traffic across lymphoid organs, enter through the bloodstream, transmigrate into tissues, and afterward, back into the blood via the lymphatic system. This migratory pattern allows for rapid mobilization of cytotoxic lymphocytes and facilitates immune surveillance. The infiltration of lymphocytes into tumors requires lymphocyte sequential interactions with endothelial cells lining tumor blood vessels (3). Beginning with low-affinity rolling along the endothelial surface mediated by selectins, lymphocytes may acquire increasing affinity to firmly bind endothelium by chemokine signalings and integrin activations, and ultimately complete the transendothelial migration process through endothelial cell junctions (4). Investigation of tumor endothelium regulatory mechanisms underlying local lymphocyte infiltration and residence has been considered critical for gaining valuable insights into immune-driven inflammatory diseases and cancers.

A growing appreciation for the immunological role played by the tumor-draining lymph nodes (TdLNs), where migratory dendritic cells (mDCs) process tumor antigens and initiate tumor-specific immunity has arisen (5, 6). Impaired TdLN functions including insufficient DC stimulation, altered cytokine signalings, and inadequate homing of lymphocytes lead to defective anti-tumor immunity. TLSs are ectopically formations that mimic the secondary lymphoid organs (SLOs), consisting of lymphocytes and antigen-presenting cells (APCs) assembled structurally. Cancer patients with TLSs tend to have more favorable outcomes and enhanced responses to immunotherapies in several studies (7, 8). As lymphoid structures formed in chronic inflammatory tumor sites, TLSs share some structural and functional features with TdLNs and are remarkable for supporting tumor immune response. TdLNs serve as important sites for the initial encounter of immune cells with tumor antigens, while TLS may form within or near the tumor, providing additional organized structures to support ongoing immune responses against the tumor. TdLNs and TLSs are interconnected components of the immune response to tumors, which contribute to local immune responses against tumors.

In the thriving era of tumor immunotherapy, attaining deep insights into lymphocyte homing and circulation is essential for acquiring a more profound comprehension of how the immune system impacts tumor development and prognosis. In this review, we introduce the multi-step migration cascades of lymphocytes tethering, rolling, adhesion, and transmigration into tumors to facilitate anti-tumor immunity. Furthermore, we point out that the deficit in lymphocyte homing represents a major contributing factor responsible for suboptimal immunotherapeutic outcomes. Meanwhile, we emphasize the importance of lymphoid aggregates in favorable tumor prognosis. TdLNs, TLSs, and even peripheral blood microenvironments that collectively influence lymphocyte homing, circulation, and residence are major targets for improving lymphocyte infiltration and recirculation in immunotherapy.

## 2 The process of lymphocytes homing and transmigration into the TME

Naïve lymphocytes that develop in primary lymphoid organs like bone marrow and thymus circulate constantly through the blood and lymphatic vessel system to SLOs including LNs, spleen, and mucosal-associated lymphoid tissues. The interaction between CCR7 and its ligands CCL19 and CCL21 is essential for the homing of naive lymphocytes to SLOs (9). Sphingosine-1-phosphate (S1P) is present in high concentrations in the blood and SLOs. The expression of S1P receptors (S1PRs) on naive lymphocytes enables them to respond to S1P gradients and migrate toward SLOs (10). Downregulation of S1PRs as a part of the tissue-resident program allows lymphocytes home and infiltrate into tissues (11). Integrins also play a pivotal role in facilitating homing of lymphocytes. Integrin bindings allow lymphocytes adhere firmly to the endothelium for transmigrating into lymphoid tissues. Naïve lymphocytes that migrate into the SLOs are primed and differentiate into effector or memory cells upon DC-induced activation and co-stimulation signalings. There exist three signal crosstalk between the lymphocyte and DC interaction. First, the antigen signaling through the binding of major histocompatibility complex (MHC) loaded with antigenic peptides to lymphocyte cell receptors mediates an antigen-specific lymphocyte activation. Second, naïve lymphocytes receive co-stimulatory signals when B7 expressed on APCs binds to CD28 on T cells, for instance. Third, instructive cytokines and chemokines like IL-12 and CCL17 secreted by activated DCs augment lymphocyte activation, with the commitment to functional proliferation and differentiation. Once lymphocytes are primed, they exit and migrate to sites of inflammation or cancers, where they recognize the same antigens that initiate the adaptive effector response.

**Figure 1:**
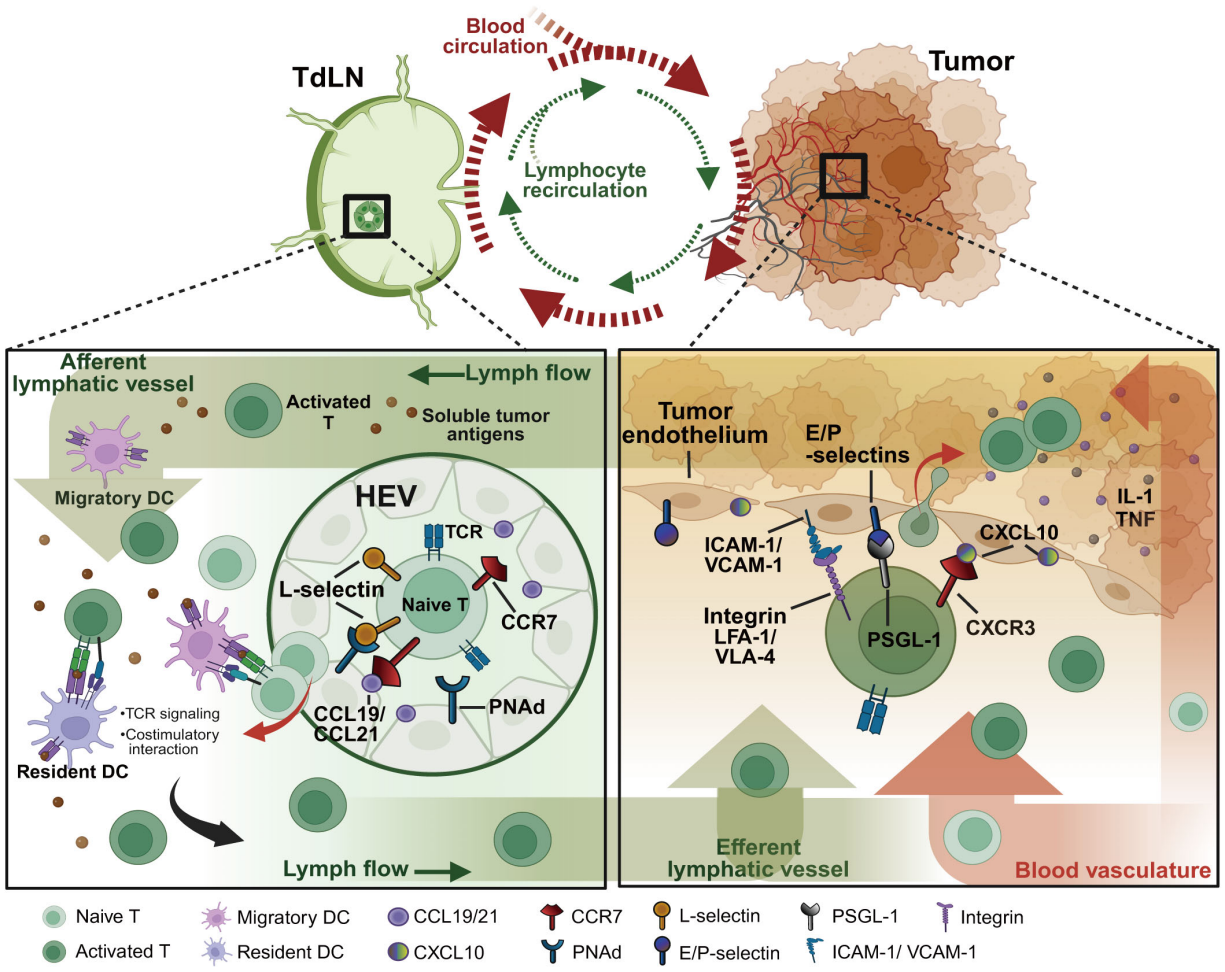
Pathways of T lymphocyte recirculation between tumor sites and TdLNs. As the tumor progresses, soluble tumor antigens traffic directly to TdLNs through the network of tumor-associated lymphatic vessels. Within the TdLNs, tumor-specific antigens stimulate migratory DCs and resident DCs to initiate anti-tumor immunity. Naïve lymphocytes expressing the homing receptors such as L-selectin and CCR7 that respectively bind PNAds and chemokines (e.g. CCL19/21) expressed on HEVs, consecutively home to TdLNs for recognizing the processed specific tumor antigens on DCs. Under the TCR and costimulatory signalings, naïve lymphocytes eventually differentiate into effector lymphocytes to migrate to tumor sites by enhanced adhesive molecule expressions. The activated integrins (e.g. LFA-1, VLA-4), selectin ligands (e.g. PSGL-1, CD43), and chemokine receptors (e.g. CXCR3, CX3CR1) on lymphocytes contribute together to lymphocyte infiltration and subsequent tumor clearance. TdLNs, tumor-draining lymph nodes; HEVs, high endothelial venules; PNAd, peripheral node addressin; PSGL-1, P-selectin glycoprotein ligand 1; VLA-4, very late antigen 4; LFA-1, leukocyte function-associated antigen 1; ICAM-1, intracellular adhesion molecule 1; VCAM-1, vascular cell adhesion molecule 1. Created with BioRender.com.

Lymphatic vessel networks allow tumor-derived signals (e.g. soluble antigens, lipids, exosomes, and nucleic acids) as well as lymphocytes to transport across LNs and tumor sites to activate anti-tumor response. During cancers, APCs like mDCs and resident DCs capture and concentrate tumor antigens that travel via lymphatic vessels from tumor sites to TdLNs (12) (**Figure 1**). Initially activated by APCs, lymphocytes in TdLNs migrate into tumor sites where they may undergo effector differentiation driven by additional co-stimulations existing in the tumor (13). In a recent study, the tumor-resident TCF-1^+^memory-like T cells were derived from the activated TdLN CD8 T cells, evidenced by TCR overlap and shared transcriptional and epigenetic features. These tumor-specific CD8 T cells with stem-like phenotypes migrate to the tumor and can only gain effector functions once infiltrating into the TME (13). Actually, the intralesional frequencies of tumor-infiltrating lymphocytes (TILs) are often below levels considered clinically beneficial. TIL infiltration is typically restricted to marginal regions rather than deeply penetrating the tumor mass (14). The process of lymphocyte transmigration into the TME consists of a regulated multi-step cascade characterized by consecutive adhesive interactions between the lymphocytes and endothelium. Accordingly, an ineffective homing of lymphocytes may be a major factor responsible for poor outcomes of immunotherapies, particularly in tumors resistant to immune-mediated lysis within such immune-insufficient infiltration microenvironments.

The transmigration process of lymphocytes into the TME involves interactions of key surface molecules between lymphocytes and tumor endothelium, such as homing receptors, chemokine receptors, addressins and chemokines. High endothelial venules (HEVs) are essential blood vessels that express high levels of addressins and adhesion molecules recognized by lymphocyte homing receptors, mediating efficient lymphocyte trafficking to lymphoid tissues (15). HEV-like tumor blood vessels that express elevated levels of the sulfated MECA-79 addressins (PNAds) participate in recruiting antigen-specific lymphocytes and are vital components of anti-tumor immune responses (3). MECA-79^+^ tumor-associated HEVs facilitate adequate lymphocyte transmigration from the peripheral into tumors. The HEV network expands during chronic inflammation in activated lymphoid structures and may undergo profound remodeling with altered vascular permeability (16). However, the mechanical forces generated by extracellular matrix (ECM) and cancer cell growth, which increase significantly in metastatic tumors and TdLNs, are potential to decrease the expression of MECA-79 on HEVs, consequently diminishing the infiltration of functional lymphocytes (17).

## 3 Adhesion signalings in regulating the transmigration program of lymphocytes into tumors

**Figure 2:**
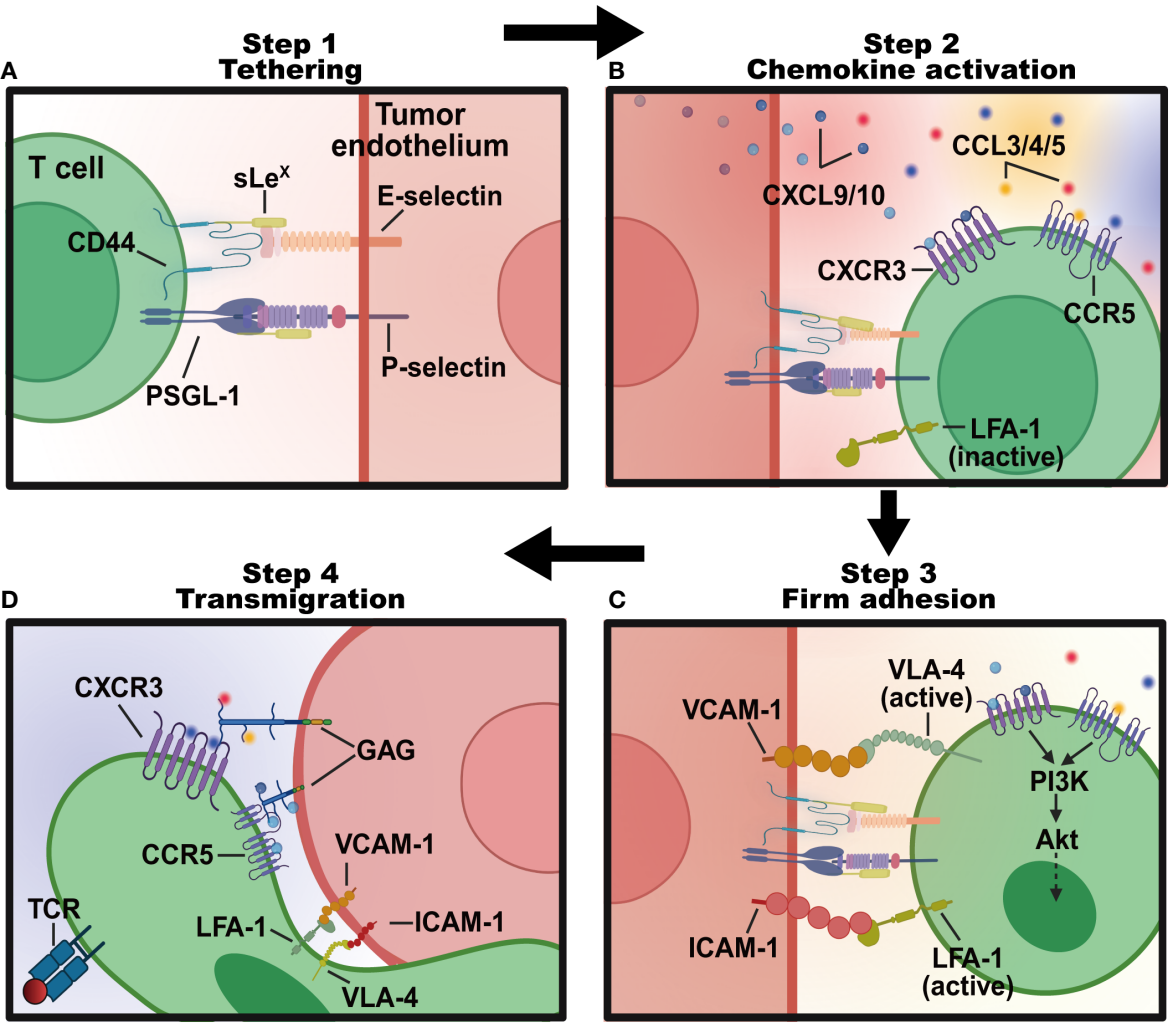
The four-step program regulating T lymphocytes transmigration into tumor sites. **(A)** Tethering: T lymphocytes roll along the endothelial cells lining tumor blood vessels in the TME, interacting weakly with E- and P-selectins expressed on cell surfaces. This process slows the T lymphocytes down and allows them to sense the microenvironment for signals of inflammation. **(B)** Chemokine activation: tethered T cells follow a chemotactic gradient of cytokines like CXCL9 and CXCL10 to exit the blood vessel and enter the inflamed tissue. **(C)** Firm adhesion: chemokines like CCL3, CCL4, and CCL5 activated T cells express integrins like LFA-1 and VLA-4 that bind strongly to adhesion molecules like ICAM-1 and VCAM-1 on endothelial cells. This stops T cells from rolling and causes them to adhere firmly. **(D)** Transendothelial migration: bound integrins trigger signaling pathways that allow the T cell to flatten out and squeeze between endothelial cells to facilitate the transmigration process of T cells into tumor sites. Created with BioRender.com.

The process of lymphocyte transmigration through the blood into tumors generally follows a four-step signaling cascade program (**Figure 2**). (1) Tethering: lymphocytes roll along and tether activated tumor endothelial cells lining specialized blood vessels through selectins. (2) Chemokine activation: inflammatory chemotactic cytokines (like CXCL9 and CXCL10) produced in the TME bind rolled lymphocytes and trigger activations of integrins. (3) Adhesion: activated integrins on lymphocytes bind to adhesion molecules like intercellular adhesion molecule-1 (ICAM-1), vascular cell adhesion molecule-1 (VCAM-1), glycosylation-dependent cell adhesion molecule-1 (GlyCAM-1), and mucosal addressin cell-adhesion molecule-1 (MadCAM-1) on tumor endothelial cells (18), mediating lymphocyte residence and firm adhesion. (4) Transendothelial migration: adhered lymphocytes crawl across the endothelium barrier and reach the extravascular solid parts of the tumor. Advanced investigation into the molecules regulating T cell recruitment will provide a deeper understanding of lymphocyte mobility (**Table 1**), informing immunotherapies designed to modulate lymphocyte infiltration.

**Table 1:** Crucial determinants for regulating TILs homing and transmigration.

Molecules	Examples	Functions	Potential therapeutic strategies
Chemokines and chemokine receptors	CXCR1-CXCL1, CXCR2-CXCL8, CCR1-CCL3/5, CCR2-CCL2, etc.	Promote TILs homing and recruitment into tumors	TIL homing agonists including chemokine receptor agonists like MCPs, MIG, MIP-1 etc., and adhesion molecule agonists like vedolizumab, crizanlizumab, efalizumab, etc.
Integrins	LFA-1, VLA-4, Mac-1, etc.		
Selectins	E/P/L-selectins		
Adhesive receptors	CEACAM-1, VAP-1, ICAM-1, VCAM-1, CD44v10, etc.	Mediate TIL adhesion to tumor cells and migration and invasion into ECM collagen gels	
Glycoproteins	TIM-1	Mediated T cell tethering and rolling on E-/P-/L-selectins	
Cytokines	IL-2, IL-7, IL-12, IL-15, etc.	Promote chemokine expressions and T cell effector	TIL Cytotoxic enhancers including checkpoint inhibitors like pembrolizumab, nivolumab, nivolumab, etc., and immunomodulatory agents like lenalidomide, thalidomide, etc.
Toll-like receptors	TLR3/7/9	Depletion of Treg cells	
Immune checkpoints	PD-1, CTLA-4, TIM-3, TIGIT, etc.	Reverse the suppression of T cell function	
Endothelins	Endothelin B receptor	Depletion of Treg cells	
Vascular growth factors	VEGF, VEGFR, etc.	Upregulate microvascular E-selectins and induce ICAM-1/VCAM-1 expression on tumor vessels	Tumor microvascular inhibitors including Ombrabulin, Vadimezan, CA4P, etc.
Stromal and myeloid cells	CAFs, myelomonocytic cells, MDSCs, and TAMs	Prevent TIL infiltration and inhibit TIL homing	Suppressive tumor microenvironment reversion like targeting MDSCs using phosphodiesterase inhibitors, CSF-1R inhibitors, etc.

TILs, tumor-infiltrating lymphocytes; LFA-1, lymphocyte function-associated antigen-1; VLA-4, very late antigen-4; Mac-1, macrophage-1 antigen; CEACAM-1, carcinoembryonic antigen cell adhesion molecule-1; VAP-1, vascular adhesion protein-1; ICAM-1, intercellular adhesion molecule-1; VCAM-1, vascular cell adhesion molecule-1; TIM, T-cell immunoglobulin and mucin domain; TLR; PD-1, programmed cell death-1; CTLA-4, cytotoxic T-lymphocyte-associated protein-4; TIGIT, T cell immunoreceptor with Ig and ITIM domains; MDSCs, myeloid-derived suppressor cells; VEGFR, vascular endothelial growth factor; CAFs, cancer-associated fibroblasts; TAMs, tumor-associated macrophages; MCPs, monocyte chemoattractant proteins; MIG, monokine-induced by gamma interferon; MIP-1, Macrophage inflammatory protein-1; CA4P, combretastatin A4 phosphate.

### 3.1 Selectins and selectin ligands in initial lymphocyte tethering

Selectins are cell membrane adhesion molecules that mediate an initial and reversible low-affinity tethering of circulating lymphocytes to endothelial cells lining postcapillary venules (19). Inflammatory mediators that are locally produced by cancer cells induce increased expression of selectins and their ligands, serving as one mechanism through which lymphocytes are recruited to sites of inflammation. Two known types of selectins are expressed by endothelial cells, P-selectins (CD62P) and E-selectins (CD62E) (20). P-selectin is stored in cytoplasmic granules within endothelial cells and resting platelets, rapidly redistributing to the cell membrane when triggered by histamine generated from thrombin and mast cells. E-selectin expression is inducible by proinflammatory cytokines such as TNF and IL-1 produced by DCs and tissue-resident macrophages in response to infections. It was illustrated that tumor-associated HEVs that co-expressed MECA-79^+^ sialomucins and E-/P-selectins were positively connected with homing and infiltration of T lymphocytes into tumors in murine tumor models (21). However, there were also reported that the high expression of P-selectin by activated platelets makes it a critical component in cancer-associated thrombosis contributing to tumor-promoting processes such as inflammation and metastasis establishment (22). In mechanism, E-/P-selectins mediate the overlapping capacity of leukocytes to roll along the vascular endothelium, and trigger an immunoreceptor-like signaling cascade that converts integrins to an affinity conformation. The slowed rolling of lymphocytes via selectins facilitates the transmigration and infiltration program (23).

By contrast, L-selectins (CD62L) exhibit constitutive expression on the surface of naive and central memory lymphocytes, including T cells and B cells. CD62L on circulating T cells serves as a peripheral blood biomarker in cancer patients for assessing immunotherapy efficacy and immune function. Memory T cells expressing CD62L are classified as central memory T cells (T_CM_) with the capacity for long-term persistence. Maintaining CD62L^+^ T_CM_ cells is crucial for durable antitumor immunity after immunotherapy. Anti-tumor therapies which are suggested to significantly suppress tumor growth were totally considered to increase the CD44^low^CD62L^hi^ memory T cells and CD44^hi^CD62L^hi^ T_CM_ cells of the tumor-infiltrating CD4 and CD8 T cells in breast cancer (24). Tumor metastasis was more likely to be enhanced by the absence of CD62L due to the defective migration of T cells and NK cells. However, the cytotoxic response was not influenced (25). The expression of CD62L also enhanced the therapeutic potential of CAR-T cells. It has been demonstrated that CAR-T cells that expanded in IL-15 preserved a less-differentiated CD62L^+^CD45RA^+^CCR7^+^stem cell memory phenotype as compared with cells cultured in IL-2, exhibiting superior antitumor responses (26). Furthermore, human-induced pluripotent stem cells (iPSCs) reprogrammed from CD62L^+^naive and memory T cells were then engineered with CD19-CARs. The iPSC CD62L^+^CAR-T cells facilitated strong antitumor activity *in vivo* and prolonged the survival of human B-cell lymphoma xenograft mouse models (27). Assessing CD62L expression on T cells has utility for immunotherapy monitoring and response prediction.

The minimal structure motif recognized by selectins is the terminal tetrasaccharide Sialyl-Lewis X (sLe^x^). The sLe^x^ is formed by post-translational modifications on various surface glycoproteins of myeloid cells and lymphocytes (28). Further sulfation of the sLe^x^ structure on galactose or N-acetylglucosamine residues increases the binding affinity of L-selectin (29). Glycosylation alterations are indicative of inflammation and tumors. P-selectin glycoprotein ligand- 1 (PSGL-1) is the well-known selectin ligand for all selectins. Binding to PSGL-1 supports the lymphocyte rolling on stimulated endothelial cells and increases the aggregation of lymphocytes (30). In induced mouse colitis models, PSGL-1 was supported to recruit effector T cell subsets by binding activated endothelial P-selectins and enhanced T cell infiltration, contributing to severe colonic tissue inflammation (31). The deficiency of PSGL-1 decreased both colonic Th1 and Th17 cytokines production (like IFN-γ, TNF, IL-17, and IL-22) because local Th17 generation is dependent on initial Th1 recruitment (31). However, in addition to its role in T cell recruitment, PSGL-1 has been shown to act as an immune checkpoint of CD8 T cells in infections and cancers (32). Similar to P-selectin, L-selectin also interacts with sulfated polysaccharides including GlyCAM-1, MAdCAM-1, and CD34 (33). GlyCAM-1 contains sLe^x^ carbohydrates and is secreted selectively on HEVs of peripheral LNs. CD34 is a transmembrane glycoprotein extensively expressed on blood vessels and hematopoietic stem cells but does not bind to L-selectin under normal conditions. CD44 is another widely glycosylated cell adhesion molecule that exhibits the strongest binding kinetics to E-selectins. The interaction between CD44 and E-selectin is proven to elongate T cell spreading and trigger lymphocyte activation (34).

Selectin-mediated tethering demonstrates rapid association and dissociation kinetics, allowing T lymphocytes to brief adhesion, and slow roll along vascular beds while remaining in proximity. Lymphocyte rolling motion is important for the subsequent steps of cell extravasation, such as firm adhesion and transendothelial migration. This slow rolling motion enables lymphocytes to scan the endothelial surface and respond to inflammatory signals, leading to their recruitment to the site of inflammation.

### 3.2 Chemokines facilitate lymphocyte firm adhesion

Several chemotactic cytokines existing in the TME stimulate lymphocyte movement and regulate the migration of lymphocytes from the blood into tumors. Produced by immune cells and several types of tumor stroma cells, chemokines activate lymphocyte intracellular signaling cascades to increase the integrin affinity and promote directed lymphocyte migration along a concentration gradient. The CXCL9, CXCL10, and CXCL11-CXCR3 axes are reported to regulate cytotoxic lymphocyte migration and mediate a tumor suppression response. CXCL9, CXCL10, and CXCL11 are mainly secreted by myeloid lineages and tumor cells in the TME responding to IFN-γ, and this process is synergistically enhanced by TNF-α (35). The expression of CXCL9 by tumor-associated macrophages regulates the recruitment and positioning of CXCR3^+^ CD8 T cells, underlying the clinical response to anti-PD(L)-1 treatment (36). CXCL10, also known as IFN-γ-induced protein 10, functions as a chemoattraction for multiple kinds of leukocytes. Comprehensive analyses of breast cancers revealed that the expression of CXCL10 was positively correlated with neoantigen load and infiltrating immune cells (37). CXCL11 is chemotactic for activated T cells. CXCL11 was reported to be a promising adjuvant of CAR-T therapy for glioblastoma. CXCL11-armed oncolytic adenoviruses increased infiltration of CD8 T lymphocytes, NK cells, and M1-polarized macrophages, while decreased proportions of myeloid-derived suppressor cells, Tregs, and M2-polarized macrophages were observed (38).

The chemokine axis CCL19/CCL21-CCR7 controls lymphocytes homing to TdLNs to encounter tumor antigens. Recent studies demonstrated that CCL19 expression accompanied with IL-7 promotes the migration and survival of CAR-T cells *in vivo* (39, 40). Engineered CAR-T cells co-expressing IL-7 and CCL21 exhibited increased anti-tumor efficacy due to significantly improved survival and infiltration of both the CAR-T cells and DCs in the TME (41). CX3CL1 is a crucial chemokine for recruiting TILs and high expression levels of CX3CL1 correlate with positive prognosis in colorectal, breast, and lung cancers (42). The distinct biological effects of CX3CL1 are mediated through its sole receptor CX3CR1, which is expressed mainly by CD8 T cells, NK cells and B cells. CXCL13 is the B cell and TFH cell chemotaxis. It contributes to germinal center (GC) formation and lymphoid structure development. Treg cells and myeloid cells rely on chemokines like CCL17, CCL22, and CCL28 to accumulate in tumors and suppress effector immune responses (43). Inhibition of these pathways may overcome tumor resistance. Chemokines like CXCL12 help maintain localized niches of cancer stem cells (CSCs) which have immunosuppressive effects through factors like IL-10 and TGF-β, leading to therapy resistance (44). Not only recruited Treg cells, CXCL12 was also self-secreted by CSCs to strongly induce cancer cell migration from the primary tumor, inhibiting possible interactions with cytotoxic T cells (44).

### 3.3 Integrins and extracellular matrix molecules in lymphocyte transmigration

Integrins facilitate firm adhesion of lymphocytes to activated endothelium and promote lymphocyte migration from blood into tissues. Lymphocyte-specific integrins involving LFA-1 and VLA-4 (α4β1) bind to ICAM-1 and VCAM-1 respectively with high affinity, not only serving as adhesive signalings for lymphocyte extravasation, but also crucial for lymphocyte communication (45, 46). Gene expression profiles of human melanoma samples with activated LFA-1 and VLA-4 expression identified improved chemokine expression like CCL4, CCL20, and CXCL12, increasing more than 6-fold CD8 effector T cell subsets and 3-fold cDC2 cells infiltration, and facilitating the remodeling of the immune cell landscape in the TME (47). In the “cold” melanoma model, the activation of LFA-1 and VLA-4 facilitated the preferential infiltration of tumor-specific T cells and reversed the T cell-exclusionary TME, improving the antitumor response synergized with CTLA-4 blockade (47). *In vivo* migration assays demonstrated that the migration of LFA-1-deficient donor lymphocytes from peripheral tissues into LNs was significantly reduced as compared to wild-type donor lymphocytes. Furthermore, the number of memory T cells in LNs was also significantly decreased in the absence of ICAM-1 or LFA-1 (48). During human acute infections, the Tbet^+^CD11c^+^ marginal zone B cells gained GC-independent memory properties depending on integrins LFA-1 and VLA-4 for efficient splenic recirculation (49). Besides LFA-1 and VLA-4, the integrin α1 subunit, also known as CD49a, is being increasingly used as a marker for lung-homing T cells (50). CD49a enables more selective trafficking of T cells into respiratory and reproductive tissues, supporting a critical role for integrins in the control of lymphocyte migration. Lymphocyte integrin α4β7 binds to MAdCAM-1 for migration into gut-associated lymphoid tissues. α4β7 can also bind VCAM-1 to regulate T cell trafficking to the inflammatory skin (51).

Nevertheless, some integrins are emerging therapeutic targets for cancer immunotherapy. Integrin activation has also been shown to enhance TGF-β expression and suppress the cytotoxic CD8 T cell response to cancer cells (52). Integrin avβ6 drove the expression of TGF-β from infiltrating lymphocytes in triple-negative breast cancer (53). What’s more, integrin αvβ3 positively regulated the expression of immune checkpoint molecules PD-L1, which impairs the effector function of T cells (54). Additionally, it is reported that TNF-α and hypoxia-inducible factor upregulate the expression of integrin and their ligands on tumor endothelial cells to promote metastasis (55, 56).

### 3.4 Cellular factors in lymphocyte recruitment

Emerging evidence indicates that DCs are critical in reprogramming lymphocytes homing in the adaptive immune system (50). DCs shape the antigen imprinting of tissue-specific T cells and the transduced co-stimulation signals impact the adhesion properties of newly primed T cells. Activation signalings from the TCR and CD28 rapidly trigger the expressions of tissue-adhesion molecules, such as P- and E-selectin on T cells. It was reported that the binding of CD80 on tissue-specific DCs to CTLA-4 on T lymphocytes contributes to the LFA-1 expression and leads to T cell residence in mucosal tissues (50). It was also observed that DCs from the lung-draining mediastinal LNs mediated the imprinting of homing to lungs for T cells in mouse models (50). The imprinted T cells expressed elevated chemokine receptors CCR4 and showed an increased capacity to transmigrate into the lung, in contrast to T cells primed by DCs from other LNs (57). In a recent study, an identified CD88^-^CD1c^+^CD163^+^progenitor DC subset was found to display a distinctive ability to prime CD8 T cells that express a tissue-homing signature with the epithelial homing alpha-E integrin (CD103) (58). Studies in mouse models have demonstrated that delivery of TBX21 (encoding transcription factor T-bet) gene-modified DCs promotes Th1 and Tc1 cell infiltration and the formation of TLSs in sarcoma tumors (59). Additionally, intratumoral infusion of CCL21 over-expressed DCs was shown to increase intratumoral CD8 T cell infiltration and reduce tumor burdens in a transgenic lung cancer mouse model (60).

Tumor-associated macrophages are also proven to affect CD8 T cell infiltration. In human breast cancer, FOLR2^+^ tissue-resident macrophages (TRM) positively correlated with the infiltration of CD8 T cells and better patient survival (61). The FOLR^+^ macrophages were found to reside in a perivascular niche in the tumor stroma and were spatially associated with tumor-infiltrating CD8 T cells. Confocal live imaging indicated that FOLR2^+^ macrophages reduced the speed of CD8 T cells and established long-lasting contacts with these cells. The prolonged interactions between CD8 T cells and FOLR2^+^ macrophages promoted T cell activation and cytotoxic function (61). Besides, tumor endothelial cells are activated by cytokines like TNF-α and IL-1 secreted by TRMs, causing upregulated expressions of adhesion molecules (such as ICAM-1, VE-cadherin and junctional adhesion molecules) and chemokines (such as CCL5, CXCL8, and CXCL12), leading to increased adhesiveness of activated lymphocytes in the TME (61–63).

Tumor endothelial cells that line abnormal tumor blood vessels are barriers found to limit lymphocyte extravasation into tumors through multiple mechanisms. Developmental endothelial locus-1 (DEL-1) secreted by tumor endothelial cells bind to integrins and phosphatidylserine, suppressing the signaling cascade that mediates lymphocyte adhesion and enhancing Treg numbers and functions at mucosae (64). DEL-1 disrupts the binding interactions between LFA-1 on lymphocytes and ICAMs on endothelial cells to modulate inflammatory responses. The deficiency of DEL-1 resulted in reduced hematopoiesis but elevated neutrophil recruitment to acute inflammation in an LFA-1-dependent adhesion way. Besides, tumor endothelial cells express soluble ICAM-5, which functions as an inhibitor of LFA-1, counteracting the pro-inflammatory effects of ICAM-1 by reducing the activation of T lymphocytes (65).

## 4 The formation of TLS is a hallmark of ongoing immune activation

TLSs develop in non-lymphoid organs that normally lack organized lymphoid aggregates at inflammatory sites including tumors, chronic infections and autoimmune diseases. Robust data show that TLSs are present in the TME of most cancer types. As a prognostic and predictive factor, TLSs have drawn strong attention to investigate its role in tumors. In the following section, we will explore the major cellular components that comprise TLSs and the underlying mechanisms driving their formation within the TME.

### 4.1 The composition and major cell types exist in TLS

**Figure 3:**
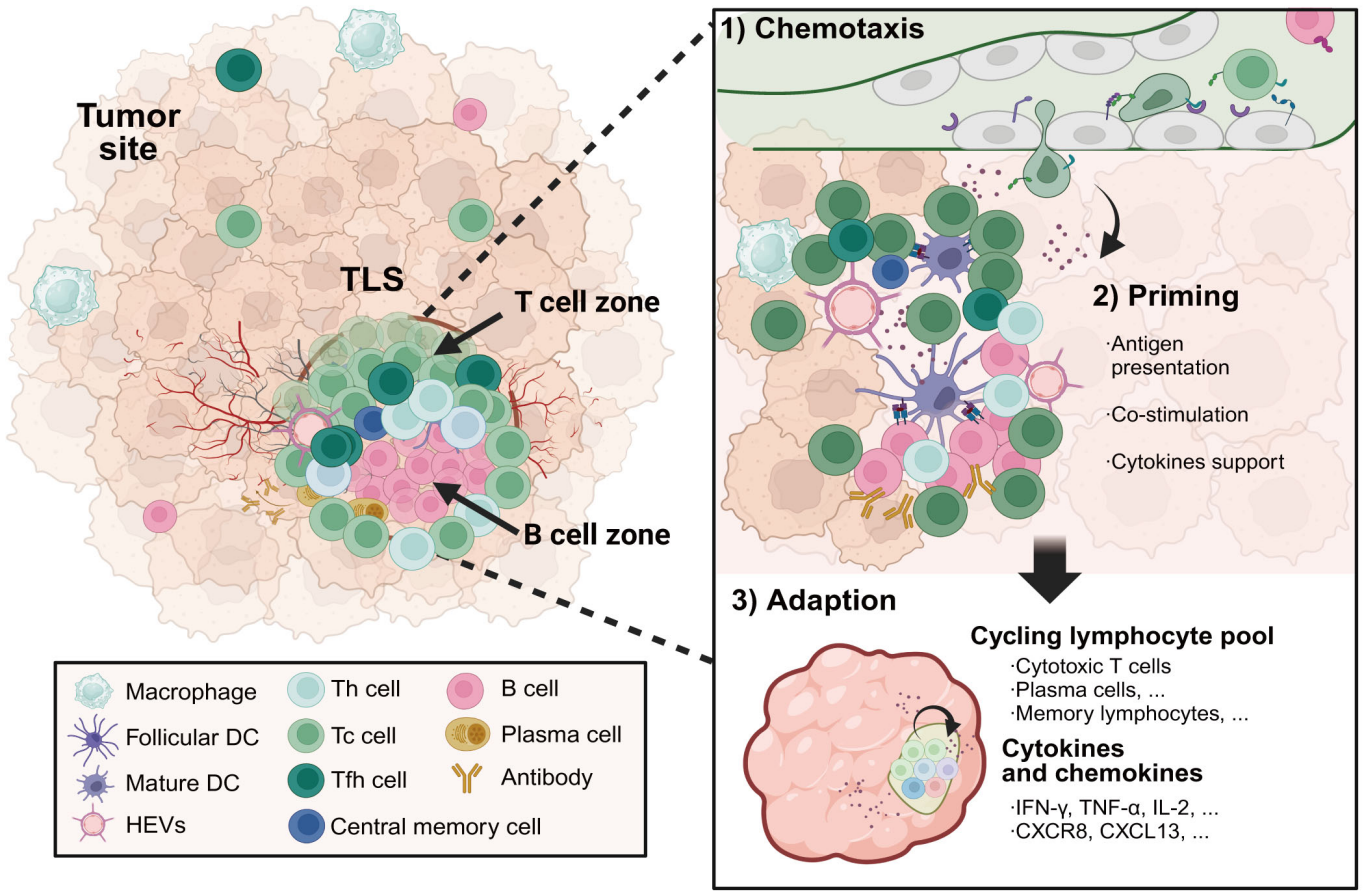
The formation and composition of TLSs in cancer. TLSs located in inflamed tumor sites serve as a niche for protecting against tumor progression. Specialized high endothelial venules and lymphatic vessels regulate the transmigration of lymphocytes through TLSs. TLSs are composed of T cell zones including CD4 helper T cells and CD8 cytotoxic T cells, as well as germinal center-like structures containing B cells and plasma cells. Priming by tumor-associated antigens presented by APCs with strong co-stimulation signalings, cytotoxic effector T cells and antibody-secreting plasma cells differentiated in TLSs facilitate *in situ* tumor destruction through distinct mechanisms by direct tumor-lysis or antibody-dependent cellular cytotoxicity mediated by macrophages and natural killer cells. Meanwhile, a subset of the activated T and B cells will develop into long-lived central memory lymphocytes, which are capable of rapidly initiating an effective immune response upon encountering the same antigens. TLSs, tertiary lymphoid structures. Created with BioRender.com.

Single-cell analyses have revealed the heterogeneous cellular composition and phenotypes of TLSs within TMEs. Commonly, TLSs are comprised of T cell zones containing CD4 helper T (Tfh) cells and CD8 cytotoxic T cells interspersed with B cell follicles harboring GCs, complemented by networks of follicular DCs (FDCs), HEVs, and lymphotoxin-expressing stromal organizer cells (**Figure 3**). Lymphocytes are the major cellular components of TLSs. Enriched for Tfh cells, CD8 cytotoxic T cells and B cells, TLS supports lymphocyte recruitment, activation, cytotoxicity maturation and memory formation. In the early phase of TLS development in ovarian cancer (66), CXCL13-producing CD4 T cells were predominantly coincident with CD4 and CD8 T cells, and it transmigrated away from T cell zones to the CD21^+^ FDCs as TLS matured in ovarian cancer. The antigen-specific LIGHT^+^CXCL13^+^IL-21^+^Tfh cells induced TLS formation and resulted in increased isotype-switched B cell responses *in vivo* (67). Studies from Gu-Trantien and her partners confirmed that CD4 Tfh cells, CD8 T cells, and B cells colocalizing in TLS, all express the CXCL13 receptor CXCR5 (68). The majority of T cells within TLSs were found to be of an effector memory phenotype in lung cancers, with few central memory T cells and naive T cells. Cytotoxic CD8 T cells have been detected in TLSs, as have CD4 T cells orientated towards a Th1 and Treg cell phenotype (69). Treg cells in TLSs suppress tumor-associated immune response. A high ratio of intratumoral Tregs to effector T cells generally predicts poor patient outcomes. What’s more, some kinds of TLSs also contain functional T follicular regulatory (Tfr) cells, which are characterized by a CD25^+^CXCR5^+^GARP^+^FOXP3^+^ phenotype and a demethylated FOXP3 gene (70). Functional Tfr cells inhibited functional Tfh activities via a glycoprotein A repetitions predominant-associated TGF-β-dependent mechanism. Indeed, the final most prominent and comprehensively analyzed anti-tumor attack within the TME is exerted by CD8 cytotoxic lymphocytes and supported by NK cells as well as IFN-γ-producing Th1 cells.

B lymphocytes are a major component of the TME, where they are predominantly associated with TLS. Unlike activated tumor-infiltrating T cells, which are primarily antigen-primed, B cells at distinct differentiation stages are detectable within the mature TLS (71). B cells are identified by surface expression of CD19 and CD20 and GC B cells intracellularly express activation-induced deaminase, CD38, and Bcl-6. The presence of CD38^+^CD138^+^plasma cells surrounding the B cell follicle is highly suggestive of the production of antibodies *in situ* (72). For lung cancer patients responding well to ICB therapy, significant enrichment sites of B cells, plasma cells and T cells were detected in a single-cell profiling study (73). One of the main effector functions associated with B cells in TLSs is the production of antigen-specific antibodies that can mark tumor cells for complement-mediated cell lysis, or antibody-dependent cellular cytotoxicity. The histological evaluation highlighted the localization of B cells within TLSs and B cell signatures were the most differentially expressed genes in the tumors of ICB responders versus non-responders (74). Although predictive biomarkers of TLSs and strategies to augment clinical response have largely focused on the T lymphocyte compartment, B cell subsets also show great potential in TLSs in the response to ICB treatment.

Among the innate immune cells, the myeloid lineages in TLSs containing DCs and macrophages play crucial roles in presenting tumor antigens to lymphocytes and mediating efficient activation. FDCs help organize B cell follicles and a network of FDCs was detected in the GC using CD21 or CD23 labeling. Activated DCs express CD83 or CD86 and mature DCs express DC-lysosome-associated membrane glycoprotein. Neutrophils express CD66b, CXCR2 and myeloperoxidase and trigger the production of pro-inflammatory cytokines that help recruit and activate lymphocytes. The NCR^+^ group 3 innate lymphoid cells (ILC3s) accumulating at the edge of TLSs in NSCLC and secreting pro-inflammatory cytokines and chemotactic factors may have lymphoid tissue inducer cell functional capability (75). Apart from the immune cells, TLSs are organized lymphoid aggregates with a network of specialized fibroblasts that share many functional and structural characteristics with SLOs. TLSs are also equipped with specialized blood vessels like HEVs that facilitate the transmigration of lymphocytes from the blood into lymphoid tissues. TLSs are enveloped by collagen and laminin basement membranes called extracellular matrix, allowing cell migration and transporting of antigens and chemokines. TLS development in murine melanoma is orchestrated by cancer-associated fibroblasts (CAFs) with characteristics of lymphoid tissue organizer cells that are induced by tumor necrosis factor receptor signaling. CAF organization into reticular networks is mediated by CD8 T cells, while CAF accumulation and TLS expansion depend on CXCL13-mediated recruitment of B cells expressing lymphotoxin-α1β2 (76).

### 4.2 The mechanism of TLS neogenesis in the TME

According to current studies, the initiation of TLS formation requires persistent inflammation, chemokine stimulation, and lymphoid organizer cell signaling activation (77, 78). Key similarities are shared between the formation of TLSs and SLOs, which provide optimized immune niches that facilitate efficient cell interactions and promote effective immune responses. Driven by the expression of the transcription factors RORγt and ID2, the lymphoid tissue inducer cells (LTi) initiate the SLO genesis in a lymphotoxin (LT) and TNF signaling-dependent manner (79, 80). LTα1β2 activates LTβ receptors (LTβRs) on epithelial cells and myeloid lineages, initiating the expressions of adhesion molecules including VCAM-1, ICAM-1, MadCAM-1, and PNAd on stromal cells (81, 82). Activated peritumoral stromal cells differentiate into specialized populations that secrete homeostatic chemokines and cytokines, including CCL19, CCL21, and CXCL13. This leads to the segregation of T and B cell areas with functional microenvironments mimicking SLOs. T cells expressing CCR7 are attracted by CCL19 and CCL21 to establish a distinct T cell zone. Moreover, B cells, through the expression of specific membrane chemokine receptors, undergo transendothelial migration into the follicle through a process regulated by the CXCR4-CXCL12 and CXCR5-CXCL13 signaling axis (83). TNF-α signaling has an important role in ectopic lymphoid structure formation. In pancreatic cancer, TNF signaling plays a crucial role in regulating the differentiation and activation of reticular networks constituted by fibroblast reticular cells (FRCs) and FDCs. Besides, Chaurio et al. recently reported that repression of Satb1 in CD4 T cells promotes Tfh cell differentiation and intratumoral TLS formation, resulting in restricted tumor growth in mice (67). Increased Tfh activity accelerated TLS-associated CD8 T cell expansion and cytotoxic functionality (84).

The development of HEVs is another factor that promotes the formation of TLSs (85). Numerous studies have demonstrated that the density of HEVs correlates with increased infiltration of CD3^+^ T cells and CD20^+^ B cells in murine cancer models (86). The HEVs present within TLSs exhibit functional similarities with those in SLOs. HEVs in TLSs express CCL21 and PNAd, highlighting their crucial roles in recruiting CD62L^+^ CCR7^+^ lymphocytes to lymphoid organs. In a Treg-depleted tumor mouse model, the HEV neogenesis was found to be dependent on TNF receptor signaling rather than LTβR signaling, implying an alternative mechanism for regulating HEV formation within lymphoid structures (87). The expression of PNAd is upregulated in response to TNF receptor activation, which is mediated by LTα3 secreted from CD8 T cells (88).

### 4.3 Contributions of TLSs to antitumor immunity

The presence of TLSs is associated with favorable prognosis in most solid malignancies like melanoma, non-small cell lung carcinoma (NSCLC) (89), renal cell cancer (RCC) and pancreatic ductal adenocarcinoma (PDAC) (90). The density of TLSs correlates with multi-subtypes of functional leukocytes such as Tfh cells, follicular B cells, and LAMP^+^ mature DCs, which generate and boost adaptive immune responses in the TME (91). Furthermore, multiple gene expression signatures related to TLSs have demonstrated favorable prognostic value, including the plasma cell signatures (IGHG, CD138, and XBP1) and the lymphoid chemokine signatures (including CCL5, CXCL9, CXCL10, and CXCL13) in human cancers (92). Amongst patients with early-stage NSCLC, approximately 70% have tumor-associated TLSs. These TLSs contain immune cells exhibiting activated phenotypes that are similar to TLSs observed after viral infection (93, 94). Two separate investigations of NSCLC patients revealed that the presence of TLSs in lesions following anti-PD-1 therapy or chemotherapy was related to extended disease-free survival and overall survival (95). However, there also been reported that intratumoral infiltration of CXCL13^+^CD8 T cells determines adverse clinical outcomes and immunoevasive microenvironment in patients with RCC (96). High-level CXCL13^+^CD8 T subset infiltration in TLS exhibited elevated exhausted markers (such as PD-1, TIM-3, and TIGIT) and descended activated markers (such as TNF-α and IFN-γ).

Emerging research indicates that TLSs play a beneficial role in the clinical response to ICB therapy, rendering these lymphoid formations appealing targets for therapeutic intervention. The combination of anti-angiogenic and immunotherapeutic approaches synergistically enhances the anti-tumor efficacy by promoting the formation of a stable vascular network that facilitates the trafficking and functional activity of effector T cells (97). Anti-vascular endothelial growth factor (VEGF) therapy synergized with anti-PD-1 and anti-CTLA-4 treatments induce a significant intratumoral effector and memory T cell infiltration, along with the *de novo* formation of intratumoral TLSs (98). Anti-VEGFR2 and anti-PD-L1 therapies increased the formation of HEVs and following TLSs in breast cancers and neuroendocrine pancreatic tumors (99). These HEVs facilitated lymphocyte infiltration and effector function activated by LTβR signaling. Moreover, LTβR agonists have been reported to induce HEV generation in resistant glioblastoma and amplify T cell cytotoxicity (99). This evidence highlights the essential of intratumoral TLSs in bolstering the effectiveness of reinvigorated antitumor responses mediated by ICBs.

## 5 Lymphoid remodeling and plasticity for lymphocyte recirculation

Lymphoid structure remodeling is characterized by lymphangiogenesis and HEV dilation, playing a critical role in facilitating tumor immune responses especially in tumor immunotherapy. Persistent exposure to tumor antigens progressively modifies and remodels LNs structurally and functionally. Tumor-induced LN remodeling is currently characterized by three principal processes: (1) lymphangiogenesis. A process involving the development of lymphatic sinuses and the increasing of lymphatic endothelial cells. VEGFs transgenically expressed in tumors activated lymphangiogenic responses in TdLNs (100). An expanded lymphatic vascular network amplifies the immune interactions in LNs but sometimes may promote dysfunctional immune responses. (2) HEV dilation and de-differentiation. The dilation of HEVs is apparent in TdLNs of patients with ductal carcinoma and becomes further exacerbated during the progression to invasive ductal carcinoma (16). Following cancer metastasizes to the LNs, significantly dilated HEVs start to lose the perivascular expressions of PNAds and CCL21 and this hampers lymphocyte recruitment (16, 17). (3) Fibrosis of the FRCs-lined conduit system (101). Fibrosis of the FRC network alters the chemokine gradients, leading to impaired lymphocyte trafficking and localization. FRCs function as structural scaffolds to organize and compartmentalize B cell follicles, T cell zones, and medullary cords within LNs. Excessive deposition of ECMs obliterates the distinct compartments and structures of FRC networks, leading to LN architectural disorganization (102).

The treatment combining ICBs led to a significant enlargement of HEVs in the mesenteric LNs. Surgical removal of TdLNs or inhibition of T cell migration through LNs can reduce the response to anti-PD(L)-1 treatment in melanoma mouse models (103, 104), suggesting the presence of lymphoid structures and recirculation of T cells to the tumor sites are crucial for immunotherapies. Recent progress in engineering lymphoid-like structures using biomaterials has led to the development of macro-scale biomaterial vaccines. Macro-scale biomaterial vaccines are developed to engineer lymphoid organs and remodel multiple types of immune cells involving DCs, lymphocytes and monocytes (105). The biomaterial scaffolds were designed to deliver tumor antigens, adjuvants or antibodies by recruiting and activating immune cells, particularly DCs, to serve as a localized immune responder in the surrounding tissues (106, 107).

## 6 Discussion

Barriers to restricting lymphocyte infiltration vary. Aberrant tumor vasculatures, altered chemokine secretion profiles, insufficient adhesive molecule expressions, and immunosuppressive cellular factors are four contributing factors. Take the limitations of CAR-T cell therapy in solid tumors as an illustration, the infiltration of CAR-T cells is influenced by dysregulated tumor vasculatures (108) and suppressive CAFs (109). CAFs impede CAR-T efficacy through elevated expressions of immune checkpoints and lymphocyte suppression molecules (110). The dysregulated vasculature system downregulates adhesive molecules required for lymphocyte transmigration and exacerbates CAR-T cell exclusion. The quantity and quality of tissue-resident lymphocytes also determine the antitumor efficacy of ICB therapies. Peripheral tissue-resident memory T cells evidenced by markers such as CD103, CD69 and CXCR6 elicited a robust cellular immunity by recruiting and activating cytotoxic CD8 T cells and NK cells in a PD-L1 blockade treatment tumor mouse model (111). As is inspired by lymphocyte recirculation and infiltration, one of the successful synergized immunotherapies might depend on the thorough acknowledgment of lymphocyte motility. Appropriate chemotactic signals for CAR-T cell trafficking and DC-based vaccinations represent improved therapeutic strategies that enhance functional lymphocyte infiltration to eradicate tumors (2, 112). APCs possess tissue-specific imprinting capability to predominantly shape the lymphocyte recirculation patterns. What’s more, the presence and abundance of TLSs may serve as valuable biomarkers in predicting patient responses to immunotherapies and clinical outcomes. Monitoring TLS formation and lymphocyte trafficking patterns may inform treatment decisions and personalized therapeutic approaches tailored to individual patients.

## Author contributions

WT: Conceptualization, Data curation, Formal Analysis, Software, Supervision, Visualization, Writing – original draft, Writing – review & editing. WW: Conceptualization, Data curation, Formal Analysis, Investigation, Validation, Writing – review & editing. GQ: Conceptualization, Formal Analysis, Resources, Software, Writing – original draft. XB: Conceptualization, Software, Supervision, Validation, Writing – review & editing. XT: Funding acquisition, Methodology, Resources, Validation, Writing – original draft. MZ: Project administration, Resources, Supervision, Validation, Writing – review & editing. YX: Methodology, Resources, Validation, Writing – original draft. YZ: Conceptualization, Funding acquisition, Project administration, Resources, Supervision, Validation, Writing – review & editing. QS: Conceptualization, Formal Analysis, Funding acquisition, Methodology, Resources, Validation, Writing – review & editing.
